# Prevalence of vitamin D inadequacy and bone turnover markers concerning bone mineral density in older persons

**DOI:** 10.25122/jml-2020-0113

**Published:** 2021

**Authors:** Abhinav Jha, Aditi Singh Tanwar, Manish Bhargava, Anil Raj, Shrimant Raman, Vishal Patil

**Affiliations:** 1. Department of Oral Pathology and Microbiology, Sarjug Dental College and Hospital, Darbhanga, Bihar, India; 2. Laxmi Dental Clinic, Varanasi, India; 3. Department of Oral Pathology and Microbiology, Manav Rachna Dental College, Faridabad, Haryana, India; 4. Department of Public Health Dentistry, Sarjug Dental College and Hospital, Darbhanga, Bihar, India; 5. Department of Prosthodontics and Crown and Bridge, Sarjug Dental College and Hospital, Darbhanga, Bihar, India; 6. Department of Prosthodontics and Crown and Bridge, Vasantdada Patil Dental College, Sangli, Maharashtra, India

**Keywords:** bone turnover marker, complete denture, relining, osteocalcin (OCN)

## Abstract

Owing to the early tooth loss in a large population, the need for removable prosthodontic therapy has widely increased. Loss of teeth can occur due to trauma, periodontal disease, dental caries, and/or oral health negligence. The removable prosthesis is preferred over other teeth replacement methods owing to its cost-effectiveness. However, due to continuous alveolar bone resorption, relining of the removable prosthesis is needed regularly. We aimed to assess the prevalence of vitamin D deficiency and bone turnover marker concerning bone mineral density. Eighty complete denture wearers either in the maxilla or in the mandible or both constituted the study sample. Before the study, all subjects were explained the procedures of the study, and informed consent was obtained before proceeding with the study. Subjects were instructed to provide an early morning sample in a fasting state as serum osteocalcin (OCN) and C-terminal telopeptide (C-Tx) have diurnal variations. Blood samples were collected, and samples were ruled out for 25-OH vitamin D, serum OCN, and C-TX. To determine the association between frequency of relining and bone turnover marker, the collected data were subjected to statistical evaluation. Significant correlations were seen between bone C-Tx, turnover markers (p<0.001), and frequency of denture relining and osteocalcin (p<0.001). No statistically significant effect of gender, vitamin D levels, age, calcium levels, vitamin D supplements, and diabetes was seen on denture relining frequency. The present study concludes that an elevated level of the bone turnover markers such as OCN and C-Tx increases the frequency of relining.

## INTRODUCTION

Due to the early tooth loss in a large number of people, the need for removable prosthodontic therapy has widely increased in the last decade. Recent literature work also suggests a less edentulous state in the near future owing to the assessment of biomarkers depicting bone turnover [1]. Loss of teeth can occur due to trauma, periodontal disease, dental caries, and/or oral health negligence. The removable prosthesis is preferred over other teeth replacement methods, given its cost-effectiveness. However, due to continuous alveolar bone resorption, relining of the removable prosthesis is needed regularly. Failure to reline dentures at particular intervals may result in ill-fitting dentures, leading to compromised mastication, esthetics, and phonetics, finally progressing to disorders of the temporomandibular joint. The activity of osteoclasts varies in denture wearers determining the relining frequency. This can be attributed to the absence of any predictor that can accurately determine osteoclastic activity rate.

Until now, there is no fixed duration of denture relining frequency in removable denture wearers. Skeletal bone loss is followed by the loss of alveolar bone [2, 3], mainly secondary to osteopenia and/or osteoporosis. Bone levels remain static and are regulated by the bone remodeling phenomenon where the combined role of osteoblastic and osteoclastic activity takes place, leading to bone formation and resorption, respectively. In subjects with bone loss, biomarkers depicting alveolar bone loss are increased, including hydroxylysine, hydroxyproline, C-terminal telopeptide, and alkaline phosphatase [4, 5]. Out of this, C-terminal telopeptide (C-Tx) and osteocalcin (OCN), along with vitamin D, are the most sensitive biomarkers and are most easily detected from the blood [6].

The present cross-sectional clinical trial hypothesizes that a significant correlation will be seen between increased relining frequency and levels of osteocalcin and C-terminal telopeptide in the serum. Also, the study aimed to characterize the relation of relining frequency and vitamin D levels in the serum.

## MATERIAL AND METHODS

Eighty complete denture wearers either in the maxilla, in the mandible, or both for at least the past year constituted the study sample. The study was conducted at the Department of Oral Pathology, Sarjug Dental College and Hospital, Darbhanga, Bihar.

Subjects having diseases affecting bone-like multiple myeloma and/or Paget's diseases or those taking bisphosphonates and glucocorticoids along with subjects with diseases like uncontrolled diabetes, liver disease, or chronic kidney disease were excluded from the study.

After this, subjects were scheduled for an appointment with dental personnel. All procedures were explained in detail to the patients. Moreover, before the study, subjects were explained about blood samples, and informed consent was obtained before starting the study. Then subjects were asked to fill a questionnaire regarding their past medical and dental history. The questionnaire also included information regarding medication, denture wearing timeline, and frequency of relining they underwent throughout the year. Denture relining history was explicitly asked. History included questions about the time and duration since they had the first denture and prosthesis changing frequency. History regarding relining due to loss of retention of denture was also recorded.

All subjects were instructed to have an early morning fasting blood sample as serum OCN and C-TX with diurnal variations. All the samples were collected and 25-OH vitamin D, serum OCN and C-Tx levels were noted.

### 25-OH vitamin D

The normal range of vitamin D in the human body is 30–100 mg/mL.

The two forms of vitamin D existing in the physiological state are vitamin D3 and vitamin D2, where D2 is obtained from dietary sources, and D3 is derived from natural sunlight. Following absorption, when vitamin D enters the circulation, its metabolization occurs in the liver, and it is converted to 25-OH-D (25-hydroxycholecalciferol). The metabolism of vitamin D partly occurs in the kidney leading to the formation of other metabolites. Being the major metabolite of vitamin D in circulation, 25-hydroxycholecalciferol constitutes the most reliable and common biomarker of vitamin D assessment using radioimmunoassay.

### Osteocalcin

The normal range is 3.2–39.6 ng/mL in males, 4.9–30.9 ng/mL in premenopausal females and 9.4–47.4 ng/mL in postmenopausal females.

Osteocalcin is synthesized by osteoblasts using vitamin K activated by 1,25-dihydroxy vitamin D. After its synthesis, osteocalcin becomes a part of the bone matrix. On commencement of the osteoclastic function, osteocalcin reaches the circulation. Hence, osteocalcin makes a reliable and easily assessed biomarker for assessing the turnover of the bone.

### C-Telopeptide

The normal range is 115–748 pg/mL in males, 112–738 pg/mL in premenopausal females and 142–1351 pg/mL in postmenopausal females.

Beta C-terminal telopeptide (β-CTX) is an osteoclastic activity turnover, acting as the biomarker for type I collagen destruction in bone by osteoclasts. The organic bone matrix is 80% comprised of collagen (type I), which is mainly formed in the bones. Remodeling occurs continuously in the bone, which leads to bone osteoclastic activity, which is caused by osteoclasts.

### Statistical Analysis

The Statistical Package for the Social Sciences (SPSS) software version 20.0.0 (IBM Corporation, HQ Armonk, New York, United States of America) was used to analyze the data. For continuous variables, means and standard deviation for the data were summarized for normally distributed data. An independent t-test was used to compare the two variables, and the ANOVA test assessed the intergroup comparisons. A p-value <0.05 defined the statistical significance. Inter-variable correlations were assessed using Pearson's chi-squared test.

## RESULTS

A total of 40 males and 40 females were involved in the study. The mean age of males and females was 69.70 and 70.70 years, respectively. The mean frequency of relining in males and females were 0.09 and 0.10 over 15 and 19 years, respectively. All the findings were mentioned in Table 1.

**Table 1:** Baseline characteristics.

Baseline characteristics		Male	Female
**Age (years) (mean±SD)**		69.70±13.09	70.70±10.30
**Frequency of reline (mean±SD)**		0.09±.08	0.10±.10
**Time (mean±SD)**		15.38±12.44	19.53±17.33
**Calcium supplements N (%)**	**No**	22 (55%)	17 (34%)
	**Yes**	18 (45%)	23 (46%)
**Vit D supplements N (%)**	**No**	19 (38%)	16 (32%)
	**Yes**	21 (42%)	24 (48%)
**Diagnosis of diabetes N (%)**	**No**	31 (62%)	26 (52%)
	**Yes**	9 (18%)	14 (35%)

Table 2 summarizes the mean levels of C-Telopeptide, osteocalcin, and Vitamin D. The relining frequency in females and males was found to be 0.10 and 0.09 per year, respectively.

**Table 2:** Effect of gender on variables.

Independent Samples Test					
Gender		N	Mean	Std.Deviation	P-value
**Vitamin D**	**Male**	40	39.48	13.21	0.27
	**Female**	40	36.52	11.49	
**C-Tx**	**Male**	40	398.68	209.58	0.69
	**Female**	40	416.18	220.62	
**OCN**	**Male**	40	23.17	12.33	0.67
	**Female**	40	23.14	13.07	
**Reline frequency**	**Male**	40	0.09	0.08	0.56
	**Female**	40	0.10	0.10	

The mean value of vitamin D, osteocalcin, and C-Telopeptide levels are depicted in Table 2. The frequency of relines in males was 0.09 per year and 0.10 in females. No statistical difference was found in the relining frequency and vitamin D, C-Telopeptide, and osteocalcin levels between males and females.

The effect of specific arch relining on levels of vitamin D, C-Tx, or osteocalcin was assessed using the ANOVA test. The results showed no statistically significant relation between arch type (mandible, maxilla, or both) and levels of the markers assessed (Table 3).

**Table 3:** Variables comparison on each arch requiring relining.

Reline Arch		N	Mean	Std. Deviation	95% Confidence Interval forMean		P-value
					Lower Bound	Upper Bound	
	**Maxilla**	12	37.57	19.89	23.59	51.54	
**Vitamin D**	**Mandible**	9	31.68	11.08	21.62	41.74	0.47
	**Both**	59	37.47	09.86	34.78	42.17	
**C-Tx**	**Maxilla**	12	366.4	247.15	208.12	524.77	0.59
	**Mandible**	9	360.23	107.53	276.076	444.50	
	**Both**	59	418.31	219.13	368.45	468.17	
	**Maxilla**	12	20.15	13.16	10.51	29.79	
**OCN**	**Mandible**	9	18.12	5.87	12.09	24.24	0.58
	**Both**	59	22.34	10.73	19.44	25.23	
**Reline frequency**	**Maxilla**	12	0.07	0.06	0.03	0.12	0.57
	**Mandible**	9	0.10	0.07	0.04	0.16	
	**Both**	59	0.10	0.09	0.08	0.13	

Pearson's correlation coefficient assessed the correlation of vitamin D level and relining frequency, as shown in Table 4. The results showed a statistically significant correlation between denture relining frequency and osteocalcin and C-Tx with a p-value of <0.001. No significant correlation was seen between vitamin D levels and relining frequency.

**Table 4:** Correlation between the variables.

Correlations		
Variable tested with the relining frequency	Pearson's correlation coefficient	P-value
**Vitamin D**	0.02	0.69
**C-Telopeptide**	0.44	<0.001*
**Osteocalcin**	0.42	<0.001*
**Age (years)**	0.01	0.83

## DISCUSSION

Ridge resorption is a continuous process leading to compromised quality and quantity of residual alveolar ridge [7]. Major resorption of the alveolar ridge is seen between 3 months and 6 months, after which residual ridge resorption decreases [8]. Resorption of the alveolar ridge is carried out by the osteoclasts, and depending on their activity, the resorption pattern differs in different individuals, at different life stages, and at various sites in one individual [9, 10]. Osteoclastic activity has different rates in two arches with higher resorption in the mandible compared to the maxilla [11, 12]. Osteoblastic activity and ridge resorption are governed by various systemic and local factors leading to alteration in the structure of the arches [13–16]. The osteoclastic activity plays a key role in denture support loss leading to unstable and ill-fitting dentures demanding frequent relining of the denture with bone resorption. This makes it unpredictable to assess the need for relining frequency. Hence, the current clinical trial aimed to determine the relationship between the relining process and biomarker levels and helps in assessing markers associated with bone resorption determining relining frequency in denture wearers.

The results of the present study showed that there exists no relationship between gender and the frequency of denture relining. When comparing alveolar bone loss in two subjects where one received placebo treatment and the other received vitamin D and calcium supplements, it was seen that alveolar bone loss was lower in subjects receiving supplements compared to subjects receiving placebo treatment [17]. However, supplements had no impact on relining frequency.

The rate of bone turnover is comparatively low in diabetic patients; however, levels of biomarkers depicting bone turnover are not affected much in diabetic patients [18]. The present study showed no statistically significant difference in non-diabetics and diabetics concerning vitamin D levels, frequency of relining, and biomarker levels.

Various studies conducted previously depict higher residual alveolar ridge resorption in the mandible compared to the maxilla [11, 12]. Hence, this was evaluated as a confounding factor. No significant difference was seen in the levels of assessed biomarkers and relining frequency in complete denture wearers, neither in the mandible or maxilla. Osteoclastic activity in either arch was not determined owing to the cross-sectional nature of the present study.

This study also showed that age did not have any effects on reline frequency. Therefore, relining frequency and biomarker levels are not dependent on age. Bisphosphonate use was not considered in the present study. However, it is considered that the use of bisphospho-nate decreases the turnover rate, and hence, levels of concerned biomarkers.

The present study established a significant correlation between osteocalcin and C-Tx levels and frequency of relining. The subjects requiring relining frequently showed higher levels of assessed bone biomarkers in the blood. Comparing osteocalcin and C-Tx, a more significant correlation was seen with C-Tx. This can be attributed to the direct relation of C-Tx to bone resorption and osteoclast function compared to osteocalcin, which is linked to osteo-blasts. The study assessed the osteoclastic activity pattern using bone marker levels, further evaluating the needed frequency of denture relining. This may help in deciding the needed frequency of relining and other teeth replacement options chosen by subjects and clinicians. The subjects that need repeated and early relining can choose other replacement options like implants in place of removable dentures, which may decrease relining need and help in alveolar bone preservation [19, 20].

Therefore, more longitudinal trials are needed to establish the cut-off values of the marker to determine risk status. Alveolar bone resorption may proceed to skeletal osteoclastic activity. Therefore, biomarker assessment could be a new and ideal method to evaluate risk factors leading to osteoporosis/osteopenia.

## CONCLUSION

Within its limitations, the present study concluded that there is a significant relationship between osteocalcin and c-telopeptide levels. The denture relining frequency was independent of factors like gender, age, or dental arch calcium levels and vitamin D supplements. More studies in the future with an increased number of subjects are required to rule out the correlation between osteocalcin and C-telopeptide levels and frequency of relining procedure required as the bone resorption process occurs continuously. The patient who requires continuous relining procedure can be offered adjunctive treatment like dental implants or the bone preservation technique to preserve the remaining part of the bone.
